# Dihydro­nium tetra­chromate(VI), (H_3_O)_2_Cr_4_O_13_

**DOI:** 10.1107/S1600536813001608

**Published:** 2013-01-23

**Authors:** Vladislav Kulikov, Gerd Meyer

**Affiliations:** a Institut für Anorganische Chemie, Universität zu Köln, Greinstrasse 6, D-50939 Köln, Germany

## Abstract

The crystal structure of (H_3_O)_2_Cr_4_O_13_ is isotypic with K_2_Cr_4_O_13_. The finite tetra­chromate anion in the title structure consists of four vertex-sharing CrO_4_ tetra­hedra and exhibits a typical zigzag arrangement. The crystal packing is stabilized by hydrogen bonds between these anions and hydro­nium cations. The two different hydro­nium cations are surrounded by nine O atoms of tetra­chromate anions, with O⋯O distances ranging between 2.866 (8) and 3.282 (7) Å.

## Related literature

The title chromate is isotypic with its potassium analogue (Casari & Langer, 2005 ▶). Löfgren (1973 ▶) and Kolitsch (2004 ▶) determined the structures of the corresponding Rb and Cs salts, respectively. For industrial applications of tetra­chromates, see: Cainelli & Cardillo (1984 ▶); Çengeloğlu *et al.* (2003 ▶). For related bond-length data, see: Casari *et al.* (2007 ▶). For cell parameters of further isolated compounds stated in the experimental procedure, see: Durif & Averbuch-Pouchot (1978 ▶) and Rahman *et al.* (2003 ▶).

## Experimental

### 

#### Crystal data

(H_3_O)_2_Cr_4_O_13_
*M* *_r_* = 454.05
Monoclinic, 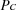

*a* = 8.9765 (13) Å
*b* = 7.6431 (8) Å
*c* = 9.3451 (14) Å
β = 91.888 (18)°
*V* = 640.80 (15) Å^3^
*Z* = 2
Mo *K*α radiation
μ = 3.37 mm^−1^
*T* = 293 K
1.0 × 0.4 × 0.2 mm

#### Data collection

Stoe IPDS I diffractometer
Absorption correction: numerical [*X-RED* (Stoe & Cie, 2001 ▶) and *X-SHAPE* (Stoe & Cie, 1999 ▶)] *T*_min_ = 0.121, *T*_max_ = 0.314
5900 measured reflections
2696 independent reflections
2497 reflections with *I* > 2σ(*I*)
*R*_int_ = 0.040

#### Refinement

*R*[*F* ^2^ > 2σ(*F* ^2^)] = 0.040
*wR*(*F* ^2^) = 0.110
*S* = 1.06
2696 reflections
174 parameters
2 restraints
Δρ_max_ = 0.69 e Å^−3^
Δρ_min_ = −0.58 e Å^−3^
Absolute structure: Flack (1983 ▶), 1212 Friedel pairs
Flack parameter: 0.53 (4)

### 

Data collection: *IPDS* (Stoe & Cie, 1997 ▶); cell refinement: *IPDS*; data reduction: *IPDS*; program(s) used to solve structure: *SIR92* (Altomare *et al.*, 1993 ▶); program(s) used to refine structure: *SHELXL97* (Sheldrick, 2008 ▶); molecular graphics: *DIAMOND* (Crystal Impact, 2012 ▶); software used to prepare material for publication: *SHELXL97*.

## Supplementary Material

Crystal structure: contains datablock(s) global, I. DOI: 10.1107/S1600536813001608/wm2714sup1.cif

Structure factors: contains datablock(s) I. DOI: 10.1107/S1600536813001608/wm2714Isup2.hkl

Additional supplementary materials:  crystallographic information; 3D view; checkCIF report

## Figures and Tables

**Fig. 1.**
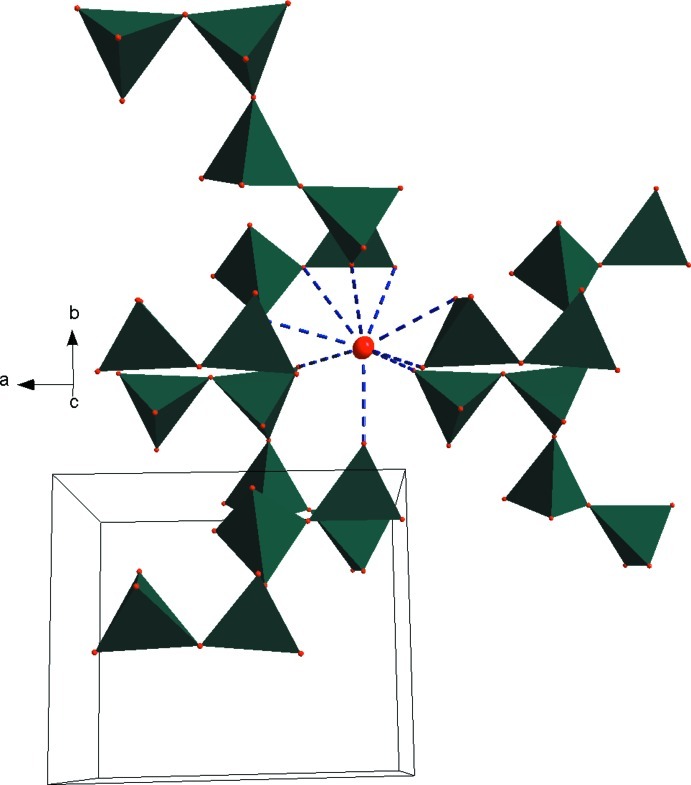
Hydrogen bonds between the hydronium ion (O1W) and the surrounding tetrachromate anions. The oxygen atom of the hydronium ion is shown with anisotropic displacement parameters at the 50% probability level. Chromate anions are shown as green-blue tetrahedra. Dashed lines denote O···O contacts between the cation and the anions.

**Fig. 2.**
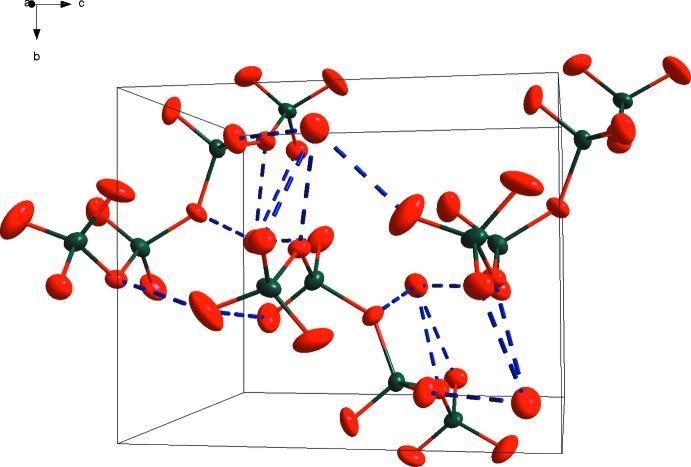
Hydrogen bonding framework within the unit cell of the compound. See Fig. 1 for legend.

## supplementary crystallographic information

### Comment

One of the characteristic properties of the elements of group 6 of the periodic table is their ability to build anionic polyoxo compounds with the corresponding elements in high oxidation states - the so called polyoxometalates. This property is profoundly exhibited by the oxoanions of the heavier elements Mo(VI) and W(VI), whereas the respective compounds of Cr(VI), i.e. polyoxochromates, are not built readily. Despite of this fact, polyoxochromates are of chemical and industrial importance due to their high oxidation potential. Accordingly, they are used as oxidants of organic compounds (Cainelli & Cardillo, 1984) and in hexavalent chromium plating (Çengeloǧlu *et al.*, 2003).

The title compound, (H_3_O)_2_Cr_4_O_13_, is only the fourth tetrachromate(VI) described and characterized by single-crystal X-ray diffraction so far. The first three are salts of alkali metals, *viz.* the potassium (Casari & Langer, 2005), the rubidium (Löfgren, 1973), and the caesium salt (Kolitsch, 2004). (H_3_O)_2_Cr_4_O_13_ was isolated from a reaction mixture containing Na_2_Cr_2_O_7_, nitric acid and a large excess of CrO_3_ along with several possibly unrelated species.

The crystal structure of (H_3_O)_2_Cr_4_O_13_ is isotypic with that of K_2_Cr_4_O_13_ (Casari & Langer, 2005) and accordingly exhibits the space group *Pc*. The cell volume of (H_3_O)_2_(Cr_4_O_13_) is 640.80 (15) Å^3^ at room temperature. It exceeds the cell volume of the potassium analogue measured at 173 K by roughly 44 Å^3^. The finite tetrachromate anion is composed of four condensed CrO_4_ tetrahedra and exhibits the typical zigzag arrangement (Figs. 1,2) described by Casari & Langer (2005). The two hydronium ions have two crystallographically different positions. They interact with nine oxygen atoms of the tetrachromate anions through hydrogen bonds. Although the H atoms of the hydronium cations could not be located, the distances between the hydronium O atoms and the surrounding tetrachromate O atoms between 2.866 (8) and 3.282 (7) Å point to moderate to weak O—H···O hydrogen bonds. These distances are quite similar to the distances between O atoms of water molecules and dichromate ions in Na_2_Cr_2_O_7_^.^H_2_O (Casari *et al.*, 2007) which indicates hydrogen bonds of typical strength for this class of compounds.

### Experimental

Na_2_Cr_2_O_7_ (26 mg, 0.1 mmol) and CrO_3_ (1.600 g, 16 mmol) were dissolved in 0.5 ml H_2_O. This solution was added to a solution of AgClO_4_ (22.5 mg, 0.1 mmol) and theobromine (18 mg, 0.1 mmol) in 16.5 ml of nitric acid. After 2.5 months crystals of (H_3_O)(ClO_4_) (Rahman *et al.*, 2003) and Ag_2_(Cr_2_O_7_) (Durif & Averbuch-Pouchot, 1978) were isolated and characterized by X-ray difractometric unit-cell determinations. Orange-red crystals of the title compound were obtained from the mother liquor after another half year.

### Refinement

The investigated crystal was racemically twinned, similarly to the potassium compound (Casari & Langer, 2005). The refined Flack paramter indicates a twin component ratio of 53 (4):47 (4). It was not possible to unambiguously locate the H atoms of the hydronium cations. They were therefore omitted from the refinement.

### Crystal data

**Table:**

(H_3_O)_2_Cr_4_O_13_	*F*(000) = 444
*M**_r_* = 454.05	*D*_x_ = 2.353 Mg m^−^^3^
Monoclinic, *P**c*	Mo *K*α radiation, λ = 0.71073 Å
Hall symbol: P -2yc	Cell parameters from 1754 reflections
*a* = 8.9765 (13) Å	θ = 1.9–28.2°
*b* = 7.6431 (8) Å	µ = 3.37 mm^−^^1^
*c* = 9.3451 (14) Å	*T* = 293 K
β = 91.888 (18)°	Block, orange-red
*V* = 640.80 (15) Å^3^	1.0 × 0.4 × 0.2 mm
*Z* = 2	

### Data collection

**Table:**

Stoe IPDS I diffractometer	2696 independent reflections
Radiation source: fine-focus sealed tube	2497 reflections with *I* > 2σ(*I*)
Graphite monochromator	*R*_int_ = 0.040
φ scans	θ_max_ = 28.1°, θ_min_ = 2.3°
Absorption correction: numerical [*X-RED* (Stoe & Cie, 2001) and *X-SHAPE* (Stoe & Cie, 1999)]	*h* = −11→11
*T*_min_ = 0.121, *T*_max_ = 0.314	*k* = −9→9
5900 measured reflections	*l* = −12→12

### Refinement

**Table:**

Refinement on *F*^2^	Secondary atom site location: difference Fourier map
Least-squares matrix: full	*w* = 1/[σ^2^(*F*_o_^2^) + (0.0842*P*)^2^] where *P* = (*F*_o_^2^ + 2*F*_c_^2^)/3
*R*[*F*^2^ > 2σ(*F*^2^)] = 0.040	(Δ/σ)_max_ < 0.001
*wR*(*F*^2^) = 0.110	Δρ_max_ = 0.69 e Å^−^^3^
*S* = 1.06	Δρ_min_ = −0.58 e Å^−^^3^
2696 reflections	Extinction correction: *SHELXL97* (Sheldrick, 2008), Fc^*^=kFc[1+0.001xFc^2^λ^3^/sin(2θ)]^-1/4^
174 parameters	Extinction coefficient: 0.083 (5)
2 restraints	Absolute structure: Flack (1983), 1212 Friedel pairs
Primary atom site location: structure-invariant direct methods	Flack parameter: 0.53 (4)

### Special details

**Table:**

Experimental. A suitable single-crystal was carefully selected under a microscope and mounted in a glass capillary. The scattering intensities were collected on an imaging plate diffractometer (*IPDS* I, Stoe & Cie) equipped with a fine focus sealed tube X-ray source (Mo Kα, λ = 0.71073 Å) operating at 50 kV and 40 mA. Intensity data for the title compound were collected at room temperature by φ-scans in 100 frames (0 < φ < 200°, Δφ = 2°, exposure time of 7 min) in the 2 Θ range 3.8 to 56.3°. Structure solution and refinement were carried out using the programs *SIR92* (Altomare *et al.*, 1993) and *SHELXL97* (Sheldrick, 1997) embedded into *WinGX* program package (Farrugia, 1999). A numerical absorption correction (*X-RED* (Stoe & Cie, 2001) was applied after optimization of the crystal shape (*X-SHAPE* (Stoe & Cie, 1999)). The last cycles of refinement included atomic positions and anisotropic parameters for all non-hydrogen atoms. Positions of hydrogen atoms were not determined. The final difference maps were free of any chemically significant features. The refinement was based on F^2^ for ALL reflections.
Geometry. All e.s.d.'s (except the e.s.d. in the dihedral angle between two l.s. planes) are estimated using the full covariance matrix. The cell e.s.d.'s are taken into account individually in the estimation of e.s.d.'s in distances, angles and torsion angles; correlations between e.s.d.'s in cell parameters are only used when they are defined by crystal symmetry. An approximate (isotropic) treatment of cell e.s.d.'s is used for estimating e.s.d.'s involving l.s. planes.
Refinement. Refinement of *F*^2^ against ALL reflections. The weighted *R*-factor *wR* and goodness of fit *S* are based on *F*^2^, conventional *R*-factors *R* are based on *F*, with *F* set to zero for negative *F*^2^. The threshold expression of *F*^2^ > σ(*F*^2^) is used only for calculating *R*-factors(gt) *etc*. and is not relevant to the choice of reflections for refinement. *R*-factors based on *F*^2^ are statistically about twice as large as those based on *F*, and *R*- factors based on ALL data will be even larger.

### Fractional atomic coordinates and isotropic or equivalent isotropic displacement parameters (Å^2^)

**Table:**

	*x*	*y*	*z*	*U*_iso_*/*U*_eq_	
Cr2	0.44108 (9)	1.10692 (11)	1.05710 (8)	0.0219 (2)	
Cr1	0.12169 (8)	0.92974 (12)	1.17627 (8)	0.0242 (2)	
Cr3	0.44219 (9)	1.44260 (12)	0.83187 (8)	0.0263 (2)	
Cr4	0.78483 (9)	1.42919 (12)	0.79827 (9)	0.0283 (2)	
O21	0.4560 (6)	0.9751 (8)	0.9295 (4)	0.0437 (12)	
O31	0.4312 (7)	1.3165 (9)	0.7003 (5)	0.0542 (13)	
O14	0.2882 (5)	1.0642 (5)	1.1542 (4)	0.0324 (9)	
O33	0.6117 (5)	1.5476 (5)	0.8324 (4)	0.0296 (8)	
O12	0.1024 (6)	0.7916 (8)	1.0473 (6)	0.0497 (12)	
O22	0.5891 (5)	1.0957 (7)	1.1545 (4)	0.0401 (11)	
O23	0.4224 (5)	1.3239 (6)	0.9907 (4)	0.0334 (9)	
O43	0.9211 (5)	1.5638 (6)	0.8196 (5)	0.0360 (10)	
O11	−0.0193 (5)	1.0612 (6)	1.1705 (5)	0.0395 (11)	
O13	0.1342 (5)	0.8307 (8)	1.3278 (6)	0.0522 (14)	
O42	0.7792 (7)	1.3587 (12)	0.6374 (7)	0.074 (2)	
O32	0.3119 (5)	1.5822 (7)	0.8197 (6)	0.0477 (13)	
O41	0.8015 (6)	1.2744 (8)	0.9121 (7)	0.0595 (16)	
O1W	0.0967 (6)	0.4158 (8)	1.0652 (6)	0.0469 (12)	
O2W	0.7959 (6)	0.9005 (7)	0.9169 (5)	0.0456 (12)	

### Atomic displacement parameters (Å^2^)

**Table:**

	*U*^11^	*U*^22^	*U*^33^	*U*^12^	*U*^13^	*U*^23^
Cr2	0.0205 (3)	0.0256 (4)	0.0197 (3)	−0.0006 (3)	0.0010 (2)	0.0020 (3)
Cr1	0.0221 (4)	0.0208 (5)	0.0296 (4)	−0.0019 (3)	0.0017 (3)	0.0052 (3)
Cr3	0.0235 (4)	0.0274 (5)	0.0278 (4)	−0.0021 (3)	0.0004 (3)	0.0068 (3)
Cr4	0.0255 (4)	0.0222 (5)	0.0374 (4)	−0.0005 (3)	0.0057 (3)	−0.0052 (3)
O21	0.047 (3)	0.060 (4)	0.0242 (18)	0.010 (2)	0.0025 (17)	−0.0114 (18)
O31	0.063 (3)	0.062 (4)	0.038 (2)	−0.024 (3)	0.0033 (19)	−0.008 (2)
O14	0.034 (2)	0.029 (3)	0.0349 (19)	−0.0062 (15)	0.0083 (16)	0.0006 (13)
O33	0.0289 (19)	0.024 (2)	0.036 (2)	−0.0003 (16)	0.0045 (15)	0.0024 (14)
O12	0.048 (3)	0.035 (3)	0.065 (3)	−0.003 (2)	−0.004 (2)	−0.017 (2)
O22	0.027 (2)	0.058 (3)	0.035 (2)	−0.0030 (18)	−0.0041 (16)	0.0082 (17)
O23	0.038 (2)	0.028 (2)	0.0344 (19)	−0.0032 (18)	0.0057 (15)	0.0107 (14)
O43	0.026 (2)	0.036 (3)	0.046 (2)	−0.0044 (15)	0.0092 (17)	0.0016 (16)
O11	0.030 (2)	0.040 (3)	0.049 (2)	0.0044 (16)	0.0039 (18)	0.0018 (17)
O13	0.038 (2)	0.061 (4)	0.057 (3)	−0.010 (2)	0.002 (2)	0.034 (3)
O42	0.059 (4)	0.092 (5)	0.071 (4)	0.006 (3)	0.007 (3)	−0.047 (4)
O32	0.029 (3)	0.057 (4)	0.057 (3)	0.006 (2)	−0.0039 (19)	0.022 (2)
O41	0.048 (3)	0.034 (3)	0.097 (4)	0.002 (2)	0.010 (3)	0.026 (3)
O1W	0.044 (3)	0.051 (3)	0.046 (2)	0.007 (2)	0.0014 (19)	−0.0030 (19)
O2W	0.043 (3)	0.049 (4)	0.044 (2)	0.009 (2)	−0.0039 (19)	0.0003 (19)

### Geometric parameters (Å, º)

**Table:**

Cr2—O21	1.570 (4)	Cr3—O31	1.563 (6)
Cr2—O22	1.588 (4)	Cr3—O32	1.584 (5)
Cr2—O14	1.701 (4)	Cr3—O33	1.720 (5)
Cr2—O23	1.776 (4)	Cr3—O23	1.754 (4)
Cr1—O13	1.606 (4)	Cr4—O41	1.595 (5)
Cr1—O12	1.607 (5)	Cr4—O42	1.596 (6)
Cr1—O11	1.615 (5)	Cr4—O43	1.606 (5)
Cr1—O14	1.831 (4)	Cr4—O33	1.836 (5)
			
O21—Cr2—O22	108.1 (3)	O32—Cr3—O33	109.7 (3)
O21—Cr2—O14	111.9 (2)	O31—Cr3—O23	110.0 (3)
O22—Cr2—O14	111.0 (2)	O32—Cr3—O23	108.3 (2)
O21—Cr2—O23	110.1 (3)	O33—Cr3—O23	110.7 (2)
O22—Cr2—O23	108.5 (2)	O41—Cr4—O42	112.2 (4)
O14—Cr2—O23	107.3 (2)	O41—Cr4—O43	109.7 (3)
O13—Cr1—O12	110.7 (3)	O42—Cr4—O43	109.5 (4)
O13—Cr1—O11	110.8 (3)	O41—Cr4—O33	108.1 (2)
O12—Cr1—O11	108.6 (3)	O42—Cr4—O33	109.3 (3)
O13—Cr1—O14	109.3 (2)	O43—Cr4—O33	108.0 (2)
O12—Cr1—O14	110.6 (2)	Cr2—O14—Cr1	147.5 (3)
O11—Cr1—O14	106.8 (2)	Cr3—O33—Cr4	121.6 (2)
O31—Cr3—O32	109.3 (3)	Cr3—O23—Cr2	140.1 (3)
O31—Cr3—O33	108.9 (3)		
